# Development and internal validation of a clinical prediction model for 1-year recurrence after first-ever ischemic stroke

**DOI:** 10.3389/fneur.2026.1820699

**Published:** 2026-04-23

**Authors:** DaYing Fan, LinHai Zhang, Rui Miao, MingLan Zhu, Cai Li, RenLi Deng, Hao Huang

**Affiliations:** 1 Nursing Department, Affiliated Hospital of Zunyi Medical University, Zunyi, China; 2 Department of Neurology, Affiliated Hospital of Zunyi Medical University, Zunyi, China; 3 Key Laboratory of Brain Function and Brain Disease Prevention and Treatment of Guizhou Province, Zunyi, China; 4 Basic Teaching Department, Zhuhai Campus of Zunyi Medical University, Zhu Hai, China

**Keywords:** clinical prediction model, internal validation, ischemic stroke, logistic regression, multiple imputation, recurrence

## Abstract

**Objective:**

Ischemic stroke (IS) recurrence remains a major contributor to poor prognosis, particularly within the 1 year after the index event. This study aimed to develop and internally validate a clinical prediction model for estimating the risk of 1-year recurrence after first-ever IS using routinely available admission data.

**Methods:**

We conducted a retrospective cohort study including consecutive patients with first-ever IS admitted to a tertiary hospital in China between January and December 2021. The primary outcome was IS recurrence within 1 year after discharge. Missing predictor data were handled using multiple imputation by chained equations. A multivariable logistic regression model was developed in the full cohort using six predictors: National Institutes of Health Stroke Scale (NIHSS) score, age, admission systolic blood pressure, uric acid, apolipoprotein A1, and neutrophil percentage. Model performance was evaluated using the area under the receiver operating characteristic curve (AUC), bootstrap internal validation with 1,000 resamples, calibration intercept, calibration slope, Brier score, and decision curve analysis.

**Results:**

Among 738 eligible patients, 96 (13.0%) experienced IS recurrence within 1 year. Higher NIHSS score, older age, higher uric acid, and higher neutrophil percentage were associated with increased recurrence risk, whereas higher apolipoprotein A1 was associated with reduced risk. Admission systolic blood pressure showed a borderline association with recurrence risk. The model demonstrated moderate discrimination, with an apparent AUC of 0.764 (95% CI 0.710–0.819). Bootstrap internal validation yielded an optimism-corrected AUC of 0.750. The bootstrap-corrected calibration intercept, calibration slope, and Brier score were 0.0058, 0.9354, and 0.0981, respectively. Decision curve analysis showed greater net benefit than the treat-all and treat-none strategies across most threshold probabilities from 0.05 to 0.60.

**Conclusion:**

We developed and internally validated a six-predictor clinical prediction model for 1-year recurrence after first-ever IS using routinely available admission variables. The model showed moderate discrimination and acceptable calibration after bootstrap correction. It may serve as a preliminary tool for risk stratification, but external validation is required before routine clinical application.

## 1 Introduction

Stroke is a leading cause of mortality and long-term disability worldwide and imposes a substantial burden on healthcare systems, particularly in low- and middle-income countries (1). Despite advances in acute management and secondary prevention strategies, a considerable proportion of patients experience recurrent ischemic events, with recurrence risk being highest soon after the index stroke and remaining substantial during the first year (2, 3). Stroke recurrence is associated with higher mortality, more severe neurological deficits, and poorer functional outcomes compared with first-ever events, underscoring the importance of early identification of patients at high risk of recurrence (4). Current approaches to secondary prevention are largely guided by population-level risk factors and etiological classifications (5). However, recurrence risk varies considerably among individuals, even within similar clinical subgroups (6). Accurate early risk stratification in routine clinical practice remains challenging. Several prediction models and risk scores for recurrent IS have been proposed, but their clinical utility is limited by heterogeneous study populations, reliance on variables not routinely available at admission, or insufficient validation in Asian populations (7). Moreover, although several tools aim to estimate 1-year recurrence risk, their evidence base remains fragmented, and external validation is often limited, underscoring the need for practical prediction models based on routinely available clinical variables.

From a pragmatic perspective, prediction models based on routinely collected admission data may offer important advantages in real-world settings (8). Commonly available variables, such as neurological severity, vital signs, and laboratory parameters, reflect multiple pathophysiological dimensions of IS, including cerebral injury burden, hemodynamic instability, metabolic stress, and inflammatory activation (9, 10). Integrating these variables into a multivariable framework may provide a more comprehensive assessment of early recurrence risk while maintaining clinical feasibility. Recent methodological advances, including penalized regression techniques, have improved the robustness of predictor selection in the presence of correlated clinical variables (11). When combined with transparent reporting standards and appropriate internal validation, these methods can support the development of clinically interpretable prediction models. Such models may facilitate individualized risk estimation while maintaining clinical interpretability (12).

In this context, we aimed to develop and internally validate a clinical prediction model for estimating the risk of 1-year IS recurrence using routinely available admission data from a real-world cohort of patients with first-ever IS. We sought to provide a preliminary tool for individualized risk stratification that may support early identification of patients at higher risk, while acknowledging that further external validation is required before routine clinical application.

## 2 Methods

### 2.1 Study design and participants

**Figure 1:**
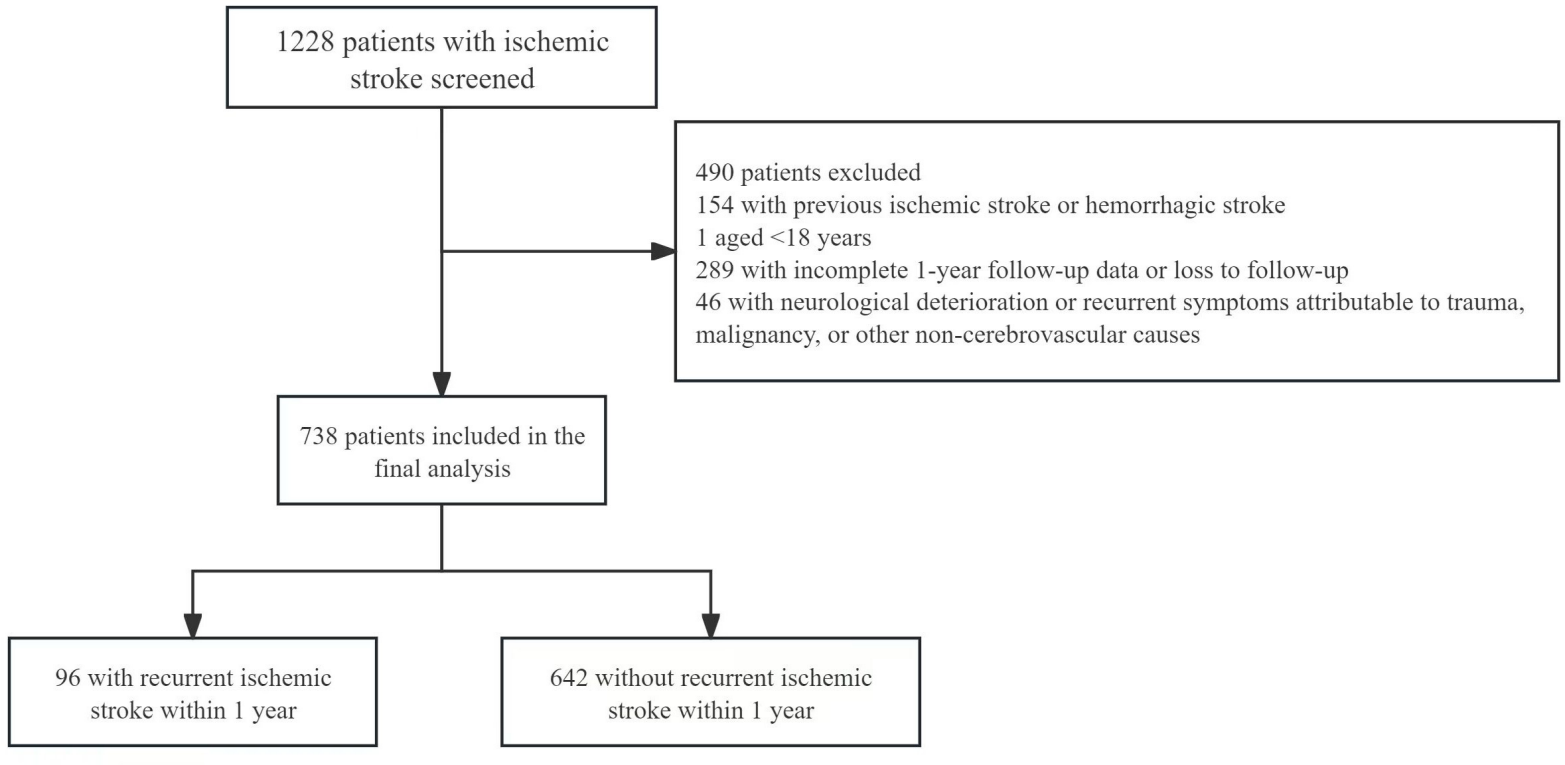
Flow diagram of patient screening, exclusion, follow-up, and final inclusion in the study.

This retrospective cohort study included consecutive adult patients with first-ever IS admitted to the Affiliated Hospital of Zunyi Medical University between 1 January and 31 December 2021. IS was diagnosed by neurologists according to the Chinese Guidelines for the Diagnosis and Treatment of Acute IS (2018 edition)

and was confirmed by neuroimaging. First-ever IS was defined as the first documented ischemic stroke event in a patient with no prior history of ischemic stroke or hemorrhagic stroke based on clinical history and available medical records. Patients were eligible if they were aged 18 years or older and had available baseline clinical information and 1-year outcome ascertainment. The primary outcome was recurrent IS within 1 year after discharge. Follow-up information was obtained from medical records and follow-up assessment. The exclusion criteria were as follows: (1) prior ischemic stroke or hemorrhagic stroke; (2) age < 18 years; (3) incomplete 1-year follow-up data; and (4) neurological deterioration or recurrent symptoms attributable to trauma, malignancy, or other non-cerebrovascular causes rather than recurrent IS. The flow diagram of patient screening, exclusion, follow-up, and final inclusion is presented in Figure 1.

### 2.2 Outcome definition

The primary outcome was fatal or non-fatal recurrent IS within 1 year after the index IS. Follow-up was conducted at 3, 6, and 12 months after discharge through outpatient visits or telephone interviews (13). Recurrent IS was defined as the occurrence of new focal neurological deficits after stabilization or resolution of index symptoms, with radiological confirmation of a new ischemic lesion on cranial CT and/or MRI, and exclusion of progressive stroke or clinical deterioration related to the index event. Outcome adjudication was independently performed by two neurologists, and disagreements were resolved by consensus.

### 2.3 Candidate predictors

Candidate predictors were prespecified based on prior literature on IS recurrence risk factors and the availability of routinely collected admission data. A total of 31 admission variables were considered, including demographic characteristics, vascular risk factors, neurological severity assessed by the National Institutes of Health Stroke Scale (NIHSS), vital signs, and laboratory parameters reflecting liver function, renal function, lipid profile, coagulation status, blood cell counts, and homocysteine levels.

### 2.4 Data collection and preprocessing

Clinical data were retrospectively extracted from the hospital electronic database. After data extraction, manual verification was independently performed by two trained data collectors to ensure data consistency. Any discrepancies were resolved by reviewing the original electronic medical records until consensus was reached. All variables were converted into analyzable formats before modeling. Binary predictors were coded as 0/1 where appropriate. The proportion of missing data for each candidate predictor was calculated and is reported in Supplementary Table S1. Missingness was low across all candidate variables, with the highest missing proportion being 2.57%. Missing predictor values were handled using multiple imputation by chained equations. Twenty imputed datasets were generated with 10 iterations. Predictive mean matching was applied to continuous variables, and logistic regression imputation was applied to binary variables. The outcome variable was not imputed but was included as a predictor in the imputation models.

### 2.5 Model development and internal validation

A total of 31 candidate predictors were initially considered for model development. Least absolute shrinkage and selection operator (LASSO) logistic regression with 10-fold cross-validation was used as a preliminary variable reduction step. Predictors with non-zero coefficients were retained as candidate variables for subsequent multivariable logistic regression analysis. The final multivariable logistic regression model retained six predictors: NIHSS score, age, admission systolic blood pressure, uric acid, apolipoprotein A1, and neutrophil percentage. To improve interpretability, uric acid was modeled per 10-μmol/L increase, and neutrophil percentage was modeled per 10-percentage-point increase. No interaction terms were prespecified. LASSO was used as a preliminary variable-reduction step to improve parsimony, whereas the final model was estimated using multivariable logistic regression to provide clinically interpretable coefficients; the potential for residual optimism associated with this hybrid strategy was subsequently assessed through bootstrap internal validation.

After multiple imputation, the final multivariable logistic regression model was fitted in the full cohort, and regression coefficients were combined across imputed datasets using Rubin's rules. Internal validation was conducted using bootstrap resampling with 1,000 repetitions to quantify optimism in model discrimination and calibration and to provide optimism-corrected performance estimates. Model discrimination was evaluated using the area under the receiver operating characteristic curve (AUC), and optimism-corrected AUC was calculated to assess potential overfitting. Calibration was assessed using the calibration intercept, calibration slope, and Brier score. A calibration plot was generated by grouping patients according to deciles of predicted probability.

### 2.6 Statistical analysis

Statistical analyses were performed using R software (version 4.5.2). Continuous variables are presented as medians with interquartile ranges and were compared using the Mann–Whitney U test. Categorical variables are presented as counts and percentages and were compared using Pearson's χ^2^ test or Fisher's exact test, as appropriate. Missing predictor data were handled using multiple imputation by chained equations, as described above. Penalized regression analyses used for preliminary variable reduction are provided in the Supplementary materials. Regression coefficients from the imputed datasets were combined using Rubin's rules. Model discrimination was evaluated using the area under the receiver operating characteristic curve (AUC), and 95% confidence intervals were estimated using the DeLong method. Internal validation was performed using bootstrap resampling with 1,000 repetitions to estimate optimism-corrected model performance. Calibration was assessed using the calibration intercept, calibration slope, Brier score, and calibration plots. Decision curve analysis (DCA) was performed to evaluate potential clinical utility by comparing the net benefit of the model with the default strategies of treating all patients or treating none across threshold probabilities of 0.05–0.60. All statistical tests were two-sided, and a *P* value < 0.05 was considered statistically significant. The study was conducted and reported in accordance with the TRIPOD statement (14).

## 3 Results

### 3.1 Baseline characteristics and correlation among candidate predictors

This retrospective study included 738 patients with first-ever IS admitted to the Affiliated Hospital of Zunyi Medical University from January to December 2021. Among them, 96 patients (13.0%) experienced recurrent IS within 1 year. The median age of the cohort was 64 years (IQR: 53–73), and 61.7% were male. The cohort had a history of cardiovascular disease in 7.0% (52/738), hypertension in 63.7% (470/738), diabetes in 18.2% (134/738), smoking in 54.9% (405/738), and alcohol use in 59.3% (438/738). Compared with patients without recurrence, those with recurrent IS had a higher prevalence of hypertension and significantly higher median NIHSS scores, older age, higher admission systolic blood pressure, higher random blood glucose, higher AST, higher urea, higher creatinine, higher LDL, higher neutrophil percentage, higher fibrinogen levels, and lower serum albumin and hemoglobin levels. No statistically significant between-group differences were observed for sex, smoking history, drinking history, diabetes, heart disease history, admission diastolic blood pressure, pulse, ALT, total cholesterol, triglycerides, HDL, uric acid, ApoA1, ApoB, homocysteine, platelet count, white blood cell count, or APTT (Table 1).

**Table 1:** Baseline characteristics of patients with and without 1-year recurrent ischemic stroke.

Variables	Category/Unit	Total (*n* = 738)	0 (*n* = 642)	1 (*n* = 96)	*P* value
Gender, *n* (%)	/				0.877
Female		283 (38.3)	245 (38.2)	38 (39.6)	
Male		455 (61.7)	397 (61.8)	58 (60.4)	
Smoking history, *n* (%)	/				0.536
No		333 (45.1)	293 (45.6)	40 (41.7)	
Yes		405 (54.9)	349 (54.4)	56 (58.3)	
Drinking history, *n* (%)	/				0.916
No		300 (40.7)	260 (40.5)	40 (41.7)	
Yes		438 (59.3)	382 (59.5)	56 (58.3)	
Hypertension, *n* (%)	/				0.010^*^
No		268 (36.3)	245 (38.2)	23 (24.0)	
Yes		470 (63.7)	397 (61.8)	73 (76.0)	
Diabetes, *n* (%)	/				0.984
No		604 (81.8)	526 (81.9)	78 (81.2)	
Yes		134 (18.2)	116 (18.1)	18 (18.8)	
Heart disease, *n* (%)	/				1
No		686 (93.0)	597 (93.0)	89 (92.7)	
Yes		52 (7.0)	45 (7.0)	7 (7.3)	
NIHSS, Median (Q1, Q3)	Points	2.00 [0.00, 5.00]	2.00 [0.00, 4.00]	4.00 [1.00, 9.50]	< 0.001^*^
Age, Median (Q1, Q3)	Years	64.00 [53.00, 73.00]	63.00 [52.00, 72.00]	71.50 [64.00, 78.00]	< 0.001^*^
Admission SBP, Median (Q1, Q3)	mmHg	138.00 [122.00, 154.00]	137.50 [120.00, 153.00]	143.50 [133.00, 161.25]	0.002^*^
Admission DBP, Median (Q1, Q3)	mmHg	83.00 [74.00, 90.00]	83.00 [74.00, 90.00]	80.50 [75.00, 90.25]	0.904
Pulse, Median (Q1, Q3)	Beats per minute	79.00 [72.00, 86.00]	78.00 [72.00, 86.00]	82.00 [74.00, 86.00]	0.161
Random BG, Median (Q1, Q3)	mmol/L	5.30 [4.65, 6.53]	5.26 [4.60, 6.28]	5.83 [4.90, 7.07]	0.002^*^
Serum albumin, Median (Q1, Q3)	g/L	38.95 [35.92, 42.30]	39.10 [36.20, 42.40]	37.95 [35.08, 40.82]	0.029^*^
AST, Median (Q1, Q3)	U/L	25.00 [21.00, 32.00]	25.00 [21.00, 31.00]	26.50 [22.00, 36.00]	0.030^*^
ALT, Median (Q1, Q3)	U/L	19.00 [14.00, 28.00]	19.00 [14.00, 28.00]	20.00 [14.00, 28.00]	0.901
Urea, Median (Q1, Q3)	mmol/L	5.19 [4.16, 6.50]	5.10 [4.10, 6.40]	5.53 [4.34, 7.72]	0.013^*^
Uric acid, Median (Q1, Q3)	μmol/L	325.50 [265.00, 391.00]	323.00 [265.00, 385.00]	344.00 [274.00, 427.75]	0.077
Creatinine, Median (Q1, Q3)	μmol/L	76.00 [63.00, 89.00]	75.00 [63.00, 88.00]	82.50 [68.00, 98.25]	0.004^*^
Total cholesterol, Median (Q1, Q3)	mmol/L	4.45 [3.72, 5.16]	4.46 [3.72, 5.19]	4.43 [3.77, 4.96]	0.269
Triglycerides, Median (Q1, Q3)	mmol/L	1.52 [1.08, 2.30]	1.54 [1.09, 2.33]	1.37 [1.04, 2.08]	0.070
HDL, Median (Q1, Q3)	mmol/L	1.10 [0.94, 1.33]	1.11 [0.95, 1.33]	1.06 [0.93, 1.31]	0.240
LDL, Median (Q1, Q3)	mmol/L	2.55 [2.11, 3.10]	2.56 [2.13, 3.12]	2.41 [1.91, 3.00]	0.020^*^
ApoA1, Median (Q1, Q3)	g/L	1.21 [1.08, 1.36]	1.22 [1.09, 1.36]	1.21 [1.01, 1.33]	0.053
ApoB, Median (Q1, Q3)	g/L	0.82 [0.69, 0.95]	0.83 [0.70, 0.95]	0.80 [0.64, 0.93]	0.095
Homocysteine, Median (Q1, Q3)	μmol/L	12.00 [7.62, 15.70]	12.00 [7.65, 15.60]	11.00 [7.00, 16.00]	0.726
Platelet count, Median (Q1, Q3)	×10^−9^/L	212.00 [169.00, 257.00]	213.00 [170.00, 257.00]	196.00 [155.75, 263.00]	0.322
Leukocyte level, Median (Q1, Q3)	×10^−9^/L	6.97 [5.64, 8.69]	6.98 [5.63, 8.63]	6.84 [5.72, 9.47]	0.340
Neutrophil percentage, Median (Q1, Q3)	%	0.66 [0.59, 0.75]	0.65 [0.59, 0.73]	0.72 [0.63, 0.82]	< 0.001^*^
Fibrinogen, Median (Q1, Q3)	g/L	3.09 [2.62, 3.90]	3.09 [2.62, 3.86]	3.38 [2.72, 4.54]	0.029^*^
APTT, Median (Q1, Q3)	Seconds	26.35 [24.90, 28.67]	26.30 [24.90, 28.50]	26.50 [24.98, 30.02]	0.368
Hemoglobin, Median (Q1, Q3)	g/L	136.00 [122.25, 148.00]	137.00 [124.00, 149.00]	130.50 [111.00, 144.00]	0.003^*^

For continuous data, values are presented as median with interquartile range. Other values are presented as number and percentage.

^*^P < 0.05 is statistically significant.

To explore interrelationships among candidate predictors, correlation analyses were performed across all 31 admission variables (Supplementary Figure S1). Neurological severity (NIHSS) showed moderate to strong positive correlations with admission systolic blood pressure, pulse rate, white blood cell count, and neutrophil percentage. Weak to moderate positive correlations were observed between NIHSS and age, admission diastolic blood pressure, urea, and fibrinogen. Strong positive correlations were detected among lipid profile markers, including total cholesterol, LDL, ApoA1, and ApoB, whereas HDL exhibited negative correlations with several atherosclerosis-related markers. Inflammatory and coagulation markers, such as white blood cell count and neutrophil percentage, correlated positively with fibrinogen. Overall, these findings highlight interdependencies among hemodynamic, inflammatory, and metabolic markers, supporting their inclusion in the predictive model for 1-year IS recurrence.

### 3.2 Predictor selection and model development

Among the 31 candidate predictors initially considered, penalized regression was used as a preliminary variable reduction step, and the detailed selection process is provided in the Supplementary materials. The final multivariable logistic regression model retained six predictors: National Institutes of Health Stroke Scale (NIHSS) score, age, admission systolic blood pressure, uric acid, apolipoprotein A1, and neutrophil percentage.

In the final model, higher NIHSS score, older age, higher uric acid, and higher neutrophil percentage were associated with an increased risk of recurrent ischemic stroke within 1 year, whereas higher apolipoprotein A1 was associated with a reduced risk. Admission systolic blood pressure showed a borderline association with recurrence risk. Specifically, the pooled odds ratios were 1.058 for NIHSS per 1-point increase (*P* = 0.002), 1.053 for age per 1-year increase (*P* < 0.001), 1.010 for admission systolic blood pressure per 1-mmHg increase (*P* = 0.061), 1.020 for uric acid per 10-μmol/L increase (*P* = 0.036), and 1.452 for neutrophil percentage per 10-percentage-point increase (*P* < 0.001). Apolipoprotein A1 was inversely associated with recurrence (OR 0.253, *P* = 0.007) (Table 2).

**Table 2:** Multivariable logistic regression results of the final six-predictor model for 1-year recurrence after first-ever ischemic stroke.

Variable	beta	SE	z	*P*	OR	CI_low	CI_high
Intercept	−8.615265984	1.342652235	−6.416602719	2.51557E-10	0.000181317	1.29915E-05	0.002530559
NIHSS (per 1–point increase)	0.056502799	0.018308283	3.086187764	0.002105592	1.058129576	1.020771215	1.096855185
Age (per 1–year increase)	0.052037073	0.010337289	5.033918604	6.06195E-07	1.053414795	1.032251805	1.075011664
Admission systolic blood pressure (per 1–mmHg increase)	0.00984365	0.005239216	1.878840346	0.060665367	1.009892258	0.999557984	1.020333376
Uric acid (per 10-μmol/L increase)	0.02005667	0.009559066	2.098183067	0.036239916	1.020259157	1.001290254	1.039587416
Apolipoprotein A1 (per 1–unit increase)	−1.372491451	0.508398191	−2.699638739	0.007115247	0.253474653	0.093413195	0.687797902
Neutrophil percentage (per 10–percentage–point increase)	0.372817508	0.108274773	3.443253665	0.000607749	1.45181937	1.173802287	1.795685274

Odds ratios (ORs), 95% confidence intervals (CIs), and *P* values of the final six predictors included in the clinical prediction model. Uric acid was modeled per 10-μmol/L increase, and neutrophil percentage was modeled per 10-percentage-point increase.

The final model was expressed as follows:


$$\begin{array}{l} logit\left(p\right)=-8.6153+0.0565\times NIHSS+0.0520\times Age+0.0098\\ \times \;Admission\;SBP+0.0201\times {Uric\;acid}_{/10}-1.3725\\ \times \;ApoA1+0.3728\times {Neutrophil\;percentage}_{/10} \end{array}$$

where *p* denotes the probability of recurrent ischemic stroke within 1 year. Uric acid was modeled per 10-μmol/L increase, and neutrophil percentage was modeled per 10-percentage-point increase.

### 3.3 Model discrimination and calibration

**Figure 2:**
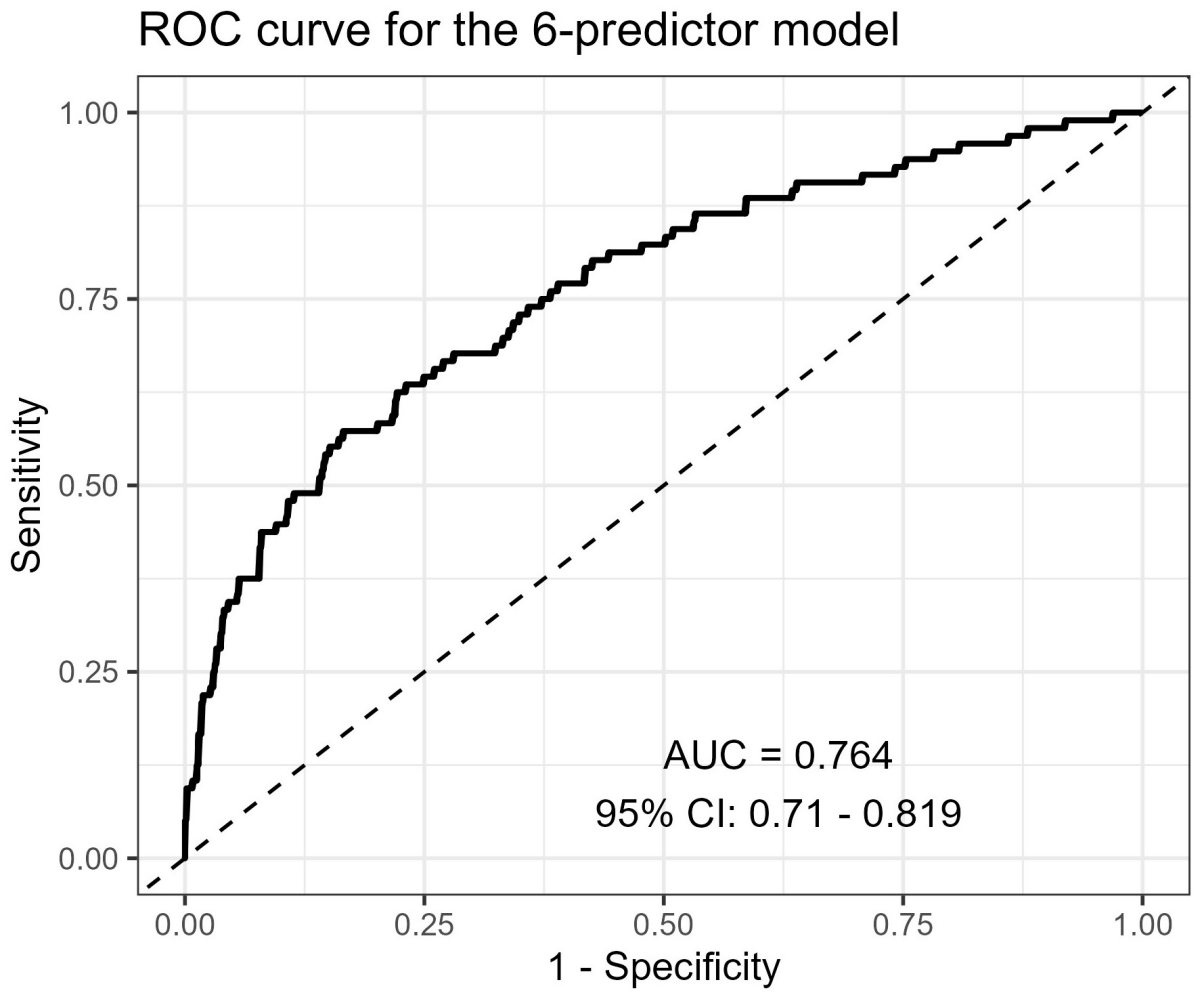
Receiver operating characteristic curve of the final six-predictor model for 1-year recurrence after first-ever ischemic stroke. The apparent area under the curve was 0.764 (95% CI 0.710–0.819).

The final six-predictor model demonstrated moderate discrimination. The apparent area under the receiver operating characteristic curve (AUC) was 0.764 (95% CI 0.710–0.819). Bootstrap internal validation with 1,000 resamples yielded an optimism-corrected AUC of 0.750, suggesting limited optimism in model performance (Figure 2).

**Figure 3:**
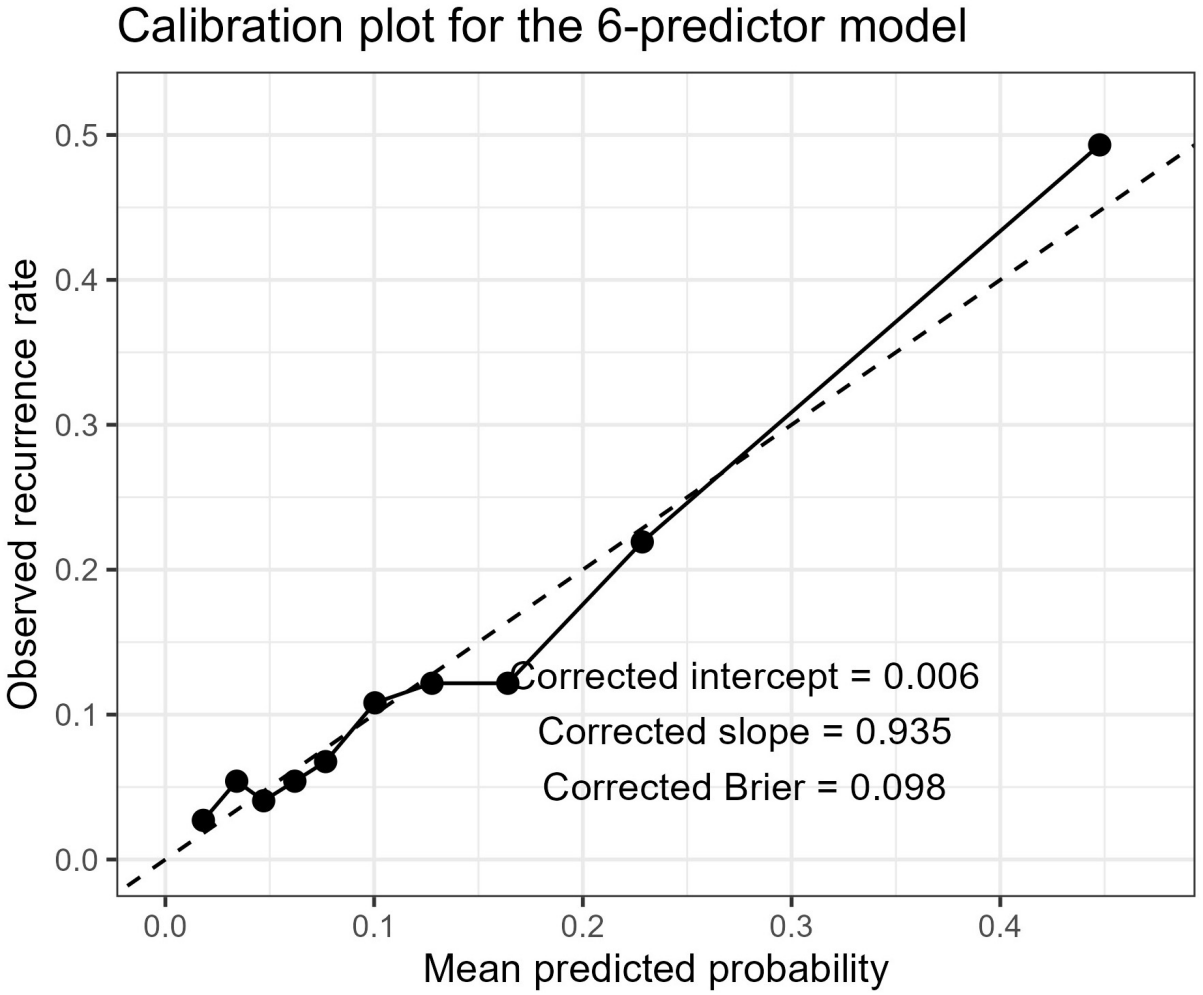
Bootstrap-corrected calibration plot of the final six-predictor model. The corrected calibration intercept was 0.0058, the corrected slope was 0.9354, and the corrected Brier score was 0.0981.

Calibration analysis showed acceptable agreement between predicted and observed recurrence risk. After bootstrap correction, the calibration intercept was 0.0058, the calibration slope was 0.9354, and the Brier score was 0.0981, indicating overall good calibration with mild overfitting. The calibration plot is presented in Figure 3.

### 3.4 Decision curve analysis

**Figure 4:**
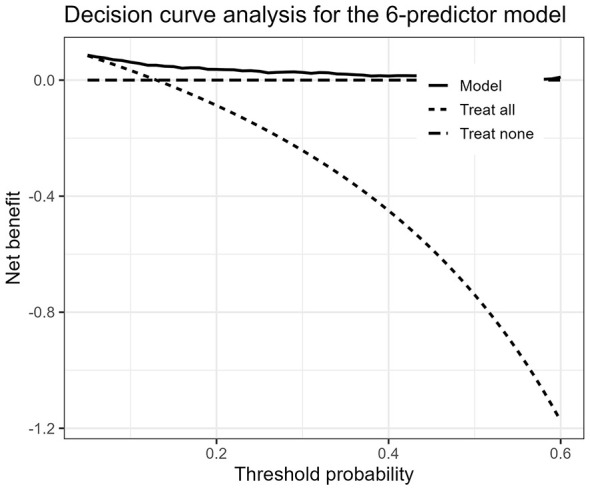
Decision curve analysis of the final six-predictor model. The model showed greater net benefit than the default strategies of treating all patients or treating none across most threshold probabilities between 0.05 and 0.60.

DCA showed that the final model provided greater net benefit than the default strategies of treating all patients or treating none across most threshold probabilities between 0.05 and 0.60 (Figure 4). The net benefit was more evident at low-to-intermediate threshold probabilities, whereas the incremental benefit became less pronounced at higher thresholds. These findings suggest that the model may have potential value for preliminary risk stratification within clinically relevant decision thresholds.

## 4 Discussion

This single-center retrospective cohort study developed and internally validated a clinical model to predict 1-year recurrence after IS. The observed recurrence level was consistent with prior reports indicating that 12-month recurrence risk remains relatively high (15), highlighting the ongoing need for more precise risk stratification despite overall improvements in secondary prevention. Among 31 routinely available admission variables initially considered, six predictors were retained in the final model: NIHSS score, age, admission systolic blood pressure, uric acid, apolipoprotein A1, and neutrophil percentage. Preliminary variable reduction was informed by penalized regression methods, and the final model was established using multivariable logistic regression (16). This hybrid strategy was chosen to balance parsimony and interpretability. However, we acknowledge that preliminary LASSO screening followed by standard logistic regression may still yield optimistic coefficient estimates, particularly in datasets with a limited number of events. We did not apply additional coefficient shrinkage in the final model, and therefore the bootstrap-corrected performance measures should be interpreted as providing reassurance regarding overall model optimism rather than eliminating all potential bias at the coefficient level. The model demonstrated moderate discrimination and acceptable calibration after internal validation, with an apparent AUC of 0.764 and an optimism-corrected AUC of 0.750. Calibration analysis showed a corrected intercept of 0.0058, a corrected slope of 0.9354, and a corrected Brier score of 0.0981, indicating only mild overfitting. Nevertheless, external validation remains necessary to confirm transportability and clinical utility across settings and case-mix (17, 18). From a PROBAST perspective, the main source of potential bias in the present study lies in the analysis domain, particularly the combination of a modest number of recurrence events, multi-step predictor selection, and the possibility of residual optimism despite bootstrap correction. Therefore, the present model should be regarded as an internally validated preliminary prediction model rather than a definitive tool for routine clinical use.

DCA further evaluated the potential clinical consequences of applying the final model across different threshold probabilities. The model provided greater net benefit than the default strategies of treating all patients or treating none across most threshold probabilities between 0.05 and 0.60, with the benefit being more evident at lower-to-intermediate thresholds. However, the incremental benefit became less pronounced at higher thresholds, indicating that the model should be interpreted cautiously and still requires external validation before clinical implementation (19).

Compared with established recurrence risk scores, traditional tools such as ESRS and SPI-II primarily focus on historical vascular risk factors and prior vascular disease, and their predictive performance for IS recurrence has been generally reported as moderate to modest (20, 21). In the Oxford Vascular Study cohort, ESRS achieved a C-statistic of approximately 0.57 (95% CI 0.54–0.60) for predicting recurrent IS (21). In addition to conventional risk factors, our model incorporated NIHSS and laboratory phenotypes reflecting inflammation and nutritional status, which may provide incremental prognostic information and partly explain the observed performance.

Among the retained predictors, NIHSS and age contributed importantly to the final model. NIHSS reflects the severity of acute neurological deficits (22) and often correlates with infarct burden, vascular lesion severity, and risk of complications; increasing age is associated with cumulative atherosclerotic burden and inflammatory activation and is linked to worse post-stroke functional outcomes (23). Correlation analyses among candidate predictors suggested interrelationships among hemodynamic, inflammatory, and metabolic markers, supporting a multifactorial view of recurrence risk (24). Clinically, these findings underscore the importance of comprehensive risk control in patients with greater neurological severity and older age.

At the laboratory level, multivariable analysis identified higher uric acid and higher neutrophil percentage as independent risk factors for recurrence, whereas higher apolipoprotein A1 was independently associated with lower risk. Uric acid and neutrophil percentage showed consistent directionality, with higher values corresponding to higher points. Uric acid may contribute to recurrence risk through oxidative stress, endothelial dysfunction, and amplification of inflammation, thereby accelerating atherosclerosis and increasing thrombotic tendency (25). Neutrophil-related indices reflect systemic inflammatory activation; inflammation-driven endothelial injury, plaque instability, and hypercoagulability may together promote recurrent events (26, 27). Conversely, apolipoprotein A1 showed an inverse effect, consistent with its role in reverse cholesterol transport and anti-inflammatory/antioxidant functions, suggesting that a more favorable lipid-metabolic and anti-inflammatory phenotype may be linked to lower recurrence risk (28, 29). Several laboratory variables that differed in univariable comparisons were not retained in the final multivariable model; this should not be interpreted as no association. Correlation analyses suggested clustering across lipid markers and other laboratory indices, and when correlated predictors are entered simultaneously, overlapping information may attenuate individual effects in multivariable analysis (30). In addition, laboratory indices may be influenced by treatment, nutritional status, and follow-up management, and effects may be diluted by heterogeneity in sampling timing (31). Prior studies have linked hypoalbuminaemia and abnormal homocysteine with adverse stroke outcomes or recurrence risk, but findings vary across populations and study designs and warrant further confirmation (32, 33). Future studies should incorporate more comprehensive information (eg, aetiological subtype, imaging burden, secondary prevention medications, and adherence) to clarify independent laboratory associations and improve interpretability and generalizability.

Because all retained predictors are routinely available from admission history and baseline laboratory testing, the model may have potential value as a preliminary tool for early risk stratification before discharge. However, this potential should be interpreted cautiously in light of the model's single-center derivation, internal validation only, and the possibility of residual optimism related to the predictor selection strategy. Several limitations should be acknowledged. First, the retrospective single-center design may introduce selection and information bias. Second, recurrence ascertainment based on local hospital records and telephone follow-up may have missed events occurring elsewhere. Third, key variables such as imaging findings, etiological classification (eg, TOAST subtype and degree of intracranial or extracranial stenosis), and post-discharge secondary prevention medications and adherence were not captured, limiting model completeness. Fourth, the binary logistic approach did not incorporate time-to-recurrence information. Finally, multicenter external validation and recalibration across regions and stroke subtypes are required to assess transportability and clinical utility.

## 5 Conclusion

This single-center retrospective study developed and internally validated a six-predictor clinical prediction model for estimating the 1-year risk of recurrent ischemic stroke using routinely collected admission clinical and laboratory variables. The final model included NIHSS score, age, admission systolic blood pressure, uric acid, apolipoprotein A1, and neutrophil percentage. It showed moderate discrimination and acceptable calibration after bootstrap internal validation. The model may serve as a preliminary tool for early risk stratification; however, because it was derived from a single-center cohort, developed using a multi-step predictor selection strategy, and underwent internal validation only, external validation in multicenter populations is required before routine clinical application.

## Data Availability

The datasets generated for and/or analyzed during the current study are not publicly available due to the privacy policy of the Affiliated Hospital of Zunyi Medical University, but are available from the corresponding author on reasonable request.
