# Supplementary material for: Development and internal validation of a clinical prediction model for 1-year recurrence after first-ever ischemic stroke
Source: Front Neurol. 2026 Apr 23;17:1820699. doi: 10.3389/fneur.2026.1820699 (PMC13149146; doi:10.3389/fneur.2026.1820699)
Supplement: Supplementary file 1 [file Supplementary_File_1.doc]

1. **Correlation among candidate predictors (Supplementary Figure S1)**
   To explore interrelationships among candidate predictors, correlation analyses were performed across all 31 admission variables. Neurological severity (NIHSS) showed moderate to strong positive correlations with admission systolic blood pressure, pulse rate, white blood cell count, and neutrophil percentage. Weak to moderate positive correlations were observed between NIHSS and age, admission diastolic blood pressure, urea, and fibrinogen. Strong positive correlations were detected among lipid profile markers, including total cholesterol, LDL, ApoA1, and ApoB, whereas HDL exhibited negative correlations with several atherosclerosis-related markers. Inflammatory and coagulation markers, such as white blood cell count and neutrophil percentage, correlated positively with fibrinogen. Overall, these findings highlight interdependencies among hemodynamic, inflammatory, and metabolic markers, supporting their inclusion in the predictive model for 1-year IS recurrence.


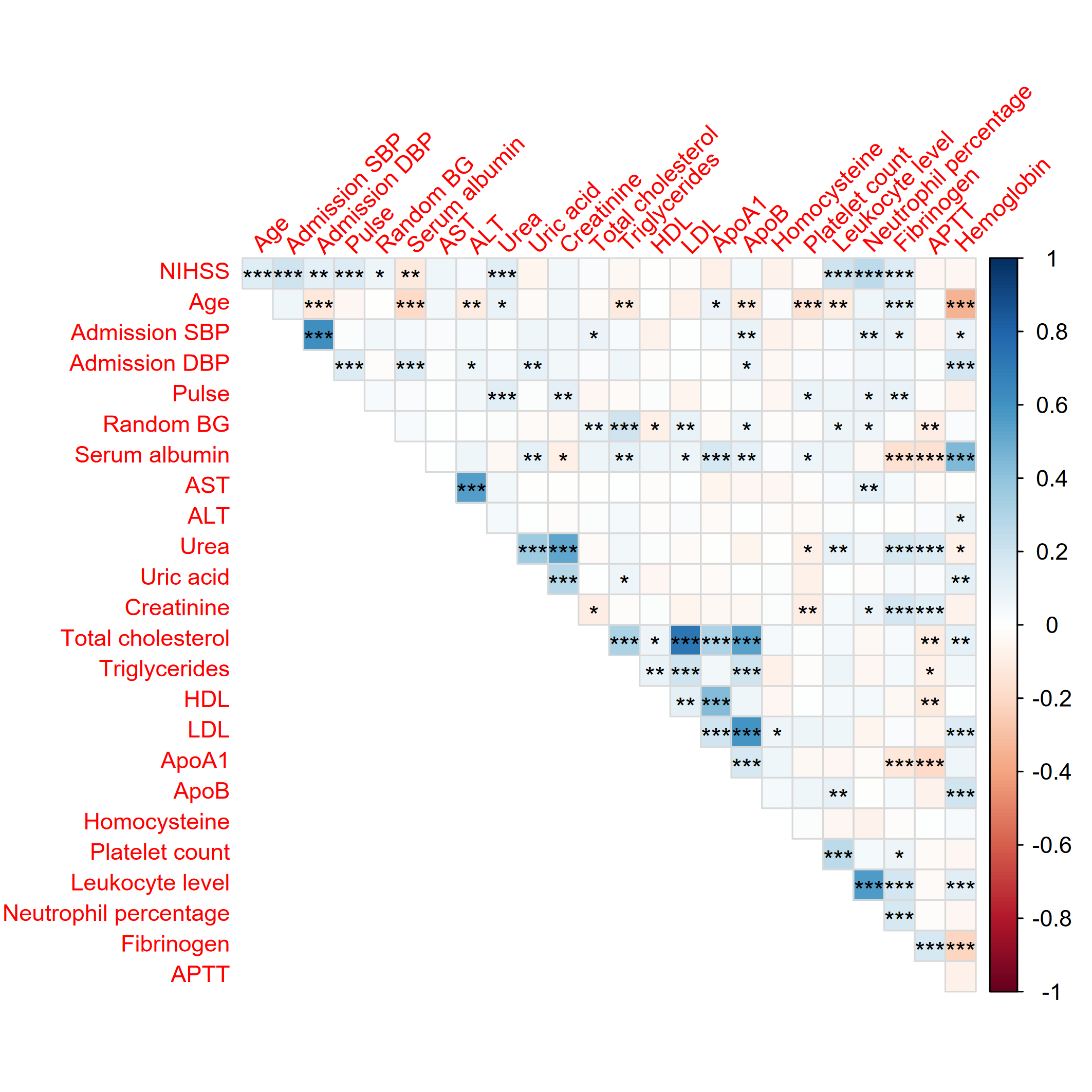
Supplementary Figure S1: Correlation heatmap of candidate predictors.

Colour indicates correlation direction and magnitude (blue, positive; red, negative); circle size reflects statistical significance. Asterisks denote *P<0.05, **P<0.01, ***P<0.001.

**2. Missing Data and Multiple Imputation**

Missingness was assessed for all 31 candidate predictors before model development. Overall, missingness was low across the dataset, and the highest proportion of missing data was 2.57%. The proportions of missing values for individual predictors are summarized in Supplementary Table S1. Because missingness was limited but non-negligible for several predictors, missing predictor values were handled using multiple imputation by chained equations. Twenty imputed datasets were generated with 10 iterations. Predictive mean matching was used for continuous variables, and logistic regression imputation was used for binary variables. The outcome variable was not imputed but was retained as a predictor in the imputation models. After imputation, complete datasets were obtained for subsequent model development and internal validation. The final multivariable logistic regression model and all validation procedures were based on the multiply imputed datasets, and regression coefficients were combined using Rubin’s rules. In the first imputed dataset, all variables had zero missing values after imputation, confirming successful completion of the imputation process.

Supplementary Table S1. Number and percentage of missing values for each candidate predictor considered in the development of the clinical prediction model.

| **variable** | **missing_n** | **missing_pct** |
| --- | --- | --- |
| fibrinogen | 19 | 2.57 |
| aptt | 19 | 2.57 |
| apoa1 | 15 | 2.03 |
| apob | 15 | 2.03 |
| triglycerides | 14 | 1.9 |
| total_cholesterol | 12 | 1.63 |
| ldl | 12 | 1.63 |
| hdl | 11 | 1.49 |
| heart_disease_history | 9 | 1.22 |
| hypertension_history | 8 | 1.08 |
| diabetes_history | 8 | 1.08 |
| serum_albumin | 5 | 0.68 |
| platelet_count | 5 | 0.68 |
| hemoglobin | 5 | 0.68 |
| alt | 4 | 0.54 |
| wbc | 4 | 0.54 |
| neutrophil_pct | 4 | 0.54 |
| nihss | 3 | 0.41 |
| uric_acid | 3 | 0.41 |
| ast | 2 | 0.27 |
| urea | 2 | 0.27 |
| creatinine | 2 | 0.27 |
| homocysteine | 2 | 0.27 |
| admission_sbp | 1 | 0.14 |
| gender | 0 | 0 |
| smoking_history | 0 | 0 |
| drinking_history | 0 | 0 |
| age | 0 | 0 |
| admission_dbp | 0 | 0 |
| pulse | 0 | 0 |
| random_bg | 0 | 0 |
| recurrence_1y | 0 | 0 |

Missingness was generally low across all candidate predictors, with the highest missing proportion being 2.57%. Missing predictor values were subsequently handled using multiple imputation by chained equations.

**3. Variable Selection and Final Model Predictors**

A total of 31 candidate variables were incorporated into the LASSO logistic regression model for feature selection. The optimal feature subset was determined through the binomial deviance plot (Supplementary Figure S2A) and coefficient trajectory plot (Supplementary Figure S2B). The binomial deviance plot showed the relationship between the regularization parameter (-log(λ)) and binomial deviance, with the number of features included at each λ value indicated at the top. The coefficient trajectory plot illustrates how the regression coefficients of each feature change with -log(λ). According to the 1SE criterion, when -log(λ) was approximately 4.0, 15 non-zero coefficients were selected. At this point, the binomial deviance stabilized, and the coefficients remained stable and non-zero, providing an optimal balance between model fit and complexity. The selected predictors included: gender, NIHSS, age, admission systolic blood pressure, serum albumin, uric acid, creatinine, triglycerides, ApoA1, homocysteine, platelet count, neutrophil percentage, APTT, fibrinogen, and hemoglobin.

These 15 predictors were then evaluated in multivariable logistic regression analysis. Six variables were retained in the final model based on overall considerations of regression results and clinical relevance: NIHSS score, age, admission systolic blood pressure, uric acid, apolipoprotein A1, and neutrophil percentage. These six predictors constitute the final clinical prediction model used for 1-year recurrence risk assessment.


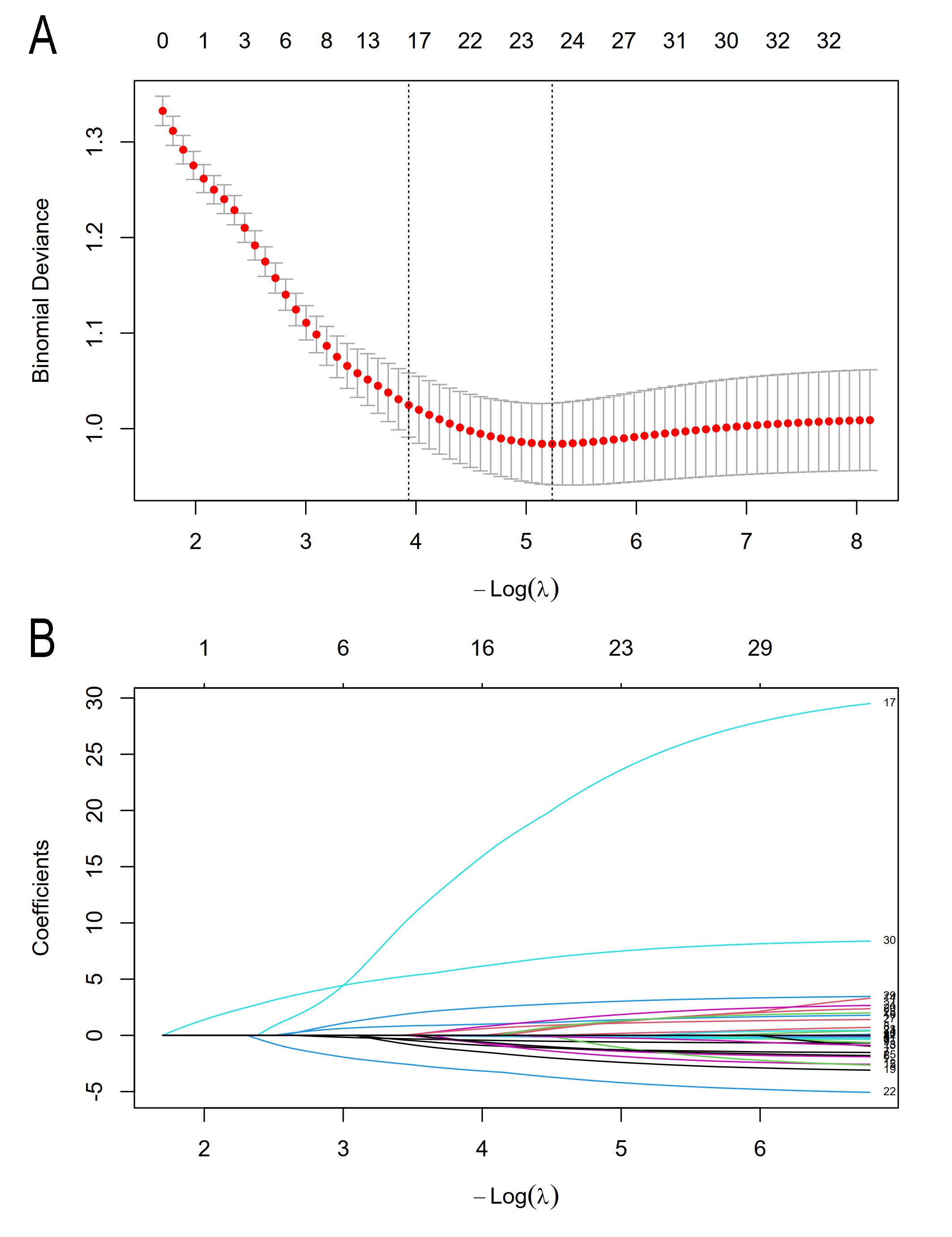
Supplementary Figure S2. LASSO feature selection for 1-year ischemic stroke recurrence. (A) Ten-fold cross-validated binomial deviance plot for selection of the regularization parameter. (B) Coefficient trajectory plot of candidate predictors across values of the regularization parameter. Predictors retained at the 1-standard-error criterion are shown.

1. Supplementary Table S2

PROBAST-based assessment of risk of bias and applicability for the present prediction model study

| Domain | Risk of bias | Applicability concern | Rationale |
| --- | --- | --- | --- |
| Participants | Low | Low | The study included consecutive adult patients with first-ever ischemic stroke from a clearly defined single-center cohort. Inclusion and exclusion criteria were prespecified, and the study population was consistent with the intended target population for early recurrence risk stratification after first-ever ischemic stroke. |
| Predictors | Low | Low | Predictors were routinely available admission variables collected before outcome ascertainment. Predictor definitions were prespecified and clinically interpretable, including neurological severity, blood pressure, and laboratory parameters commonly available in routine practice. |
| Outcome | Low | Low | The outcome was recurrent ischemic stroke within 1 year after discharge. Outcome definition was clinically explicit and required new neurological deficits together with radiological confirmation of a new ischemic lesion. Outcome adjudication was performed independently by two neurologists. |
| Analysis | High | Low | The analysis domain was judged to be at high risk of bias because the number of recurrence events was modest relative to the model development process, predictor selection involved a multi-step strategy (preliminary LASSO screening followed by multivariable logistic regression), and no additional coefficient shrinkage was applied in the final model. Although multiple imputation and bootstrap internal validation were performed, residual optimism and coefficient instability may still remain. |
| Overall | High | Low | The overall risk of bias was judged to be high, driven mainly by concerns in the analysis domain. Applicability concerns were considered low because the study population, predictors, and outcome were broadly consistent with the intended clinical use of the model. |

Footnote: The overall risk of bias was judged to be high mainly because of concerns in the analysis domain, including the modest number of events relative to the modeling process, multi-step predictor selection, and the possibility of residual optimism despite bootstrap correction. Applicability concerns were judged to be low.
